# TMBcalc: a computational pipeline for identifying pan-cancer Tumor Mutational Burden gene signatures

**DOI:** 10.3389/fgene.2024.1285305

**Published:** 2024-04-05

**Authors:** Grete Francesca Privitera, Salvatore Alaimo, Anna Caruso, Alfredo Ferro, Stefano Forte, Alfredo Pulvirenti

**Affiliations:** ^1^ Department of Clinical and Experimental Medicine, Bioinformatics Unit, University of Catania, Catania, Italy; ^2^ Department of Physics and Astronomy, University of Catania, Catania, Italy; ^3^ Istituto Oncologico del Mediterraneo (IOM) Ricerca, Viagrande, Italy

**Keywords:** personalized medicine, Tumor Mutational Burden, DNA-seq, analysis pipeline, pan-cancer

## Abstract

**Background:**

In the precision medicine era, identifying predictive factors to select patients most likely to benefit from treatment with immunological agents is a crucial and open challenge in oncology.

**Methods:**

This paper presents a pan-cancer analysis of Tumor Mutational Burden (TMB). We developed a novel computational pipeline, TMBcalc, to calculate the TMB. Our methodology can identify small and reliable gene signatures to estimate TMB from custom targeted-sequencing panels. For this purpose, our pipeline has been trained on top of 17 cancer types data obtained from TCGA.

**Results:**

Our results show that TMB, computed through the identified signature, strongly correlates with TMB obtained from whole-exome sequencing (WES).

**Conclusion:**

We have rigorously analyzed the effectiveness of our methodology on top of several independent datasets. In particular we conducted a comprehensive testing on: (i) 126 samples sourced from the TCGA database; few independent whole-exome sequencing (WES) datasets linked to colon, breast, and liver cancers, all acquired from the EGA and the ICGC Data Portal. This rigorous evaluation clearly highlights the robustness and practicality of our approach, positioning it as a promising avenue for driving substantial progress within the realm of clinical practice.

## 1 Introduction

Cancer Immunotherapy aims to activate or boost patients’ adaptive or innate immune systems to attack tumor cells. Indeed, tumor cells carry out several mechanisms to evade their recognition and elimination by T cells. Firstly, they express the programmed cell death ligand 1 (PD-L1), which binds the programmed death receptor 1 (PD1) on T cells, making them inactive. Secondly, when cytotoxic T lymphocyte antigen-4 (CTLA-4), a co-inhibitory molecule that regulates the T cell activation, interacts with its ligands (CD80 and CD86), it inhibits T cell activity promoting immunological escape. Lastly, tumor cells can lose the expression of both Major Histocompatibility Complex (MHC) classes, thus becoming invisible to the host immune system. In the last few years, it has been observed that T cell therapies and monoclonal antibodies blocking the CTLA-4 and PD1 immune checkpoints can induce durable responses across tumors. In particular, the PD-L1 ligand has been studied since it is commonly upregulated on several human solid tumors, including Melanoma, Lung, and Ovarian cancers, leading to peripheral T cell exhaustion and inhibition of apoptosis of malignant cells (Harview et al., 2014). Immune checkpoint inhibitors enhance anti-tumor T-cell activity by inhibiting immune checkpoint molecules. While the immune system plays a pivotal role in neoplastic controls, its tolerance to physiological elements must be granted by specific signals distinguishing cancer cells from normal cells. Indeed, cancer cells are recognized thanks to particular antigens such as tumor-associated antigens (TAA), over-expressed in tumor cells, and neoantigens, also called tumor-specific antigens (TSA). Usually, these antigens are not expressed by normal cells, but they are produced in tumor cells due to mutations in coding sequences. For this reason, these are ideal targets for T cell-based cancer immunotherapy. Point-mutated genes encode the majority of these neoantigens. Identifying reliable predictive factors allowing the selection of patients most likely to benefit from treatment with immunological agents is still an open challenge in oncology (Hegde and Chen, 2020; Kossai et al., 2021). Unfortunately, some patients do not respond to immunotherapy and, in addition, immunotherapy could lead to unpleasant side effects such as skin rash, colitis, hepatotoxicity, pneumonitis, endocrinopathies, and autoimmune diseases (Golshani and Zhang, 2020). The biomarkers used in cancer immunotherapy include PD-L1 and PD-L2 expression levels, microsatellite instability (MSI) status, neoantigen presence, and Tumor Mutational Load or Tumor Mutational Burden (TMB) (Shu et al., 2020).

In the past years, the most used biomarker to decide immunotherapy was immunohistochemistry’s evaluation of PD-1/PD-L1 protein expression. However, this biomarker is challenging to interpret. Hence, TMB has been considerably studied as a biomarker in recent years. TMB is defined by counting all the mutations found in a tumor sample divided by the total length of the sequenced sample in DNA Megabase (Mb). Many mutations in a tumor harbor more neoantigens, making them targets of activated immune cells. In addition, the mutation number and DNA structural alterations lead to the production of foreign proteins recognizable by the immune system. Therefore, establishing the mutational load in cancer cells would allow identifying those patients who can benefit significantly from this type of therapy compared to conventional chemotherapy treatment. However, there is no standardized specific value to decide which is a high TMB (H-TMB) or a low TMB (L-TMB) level (Lawrence et al., 2013; Stenzinger et al., 2019). Few commercial and free pipelines have been implemented to analyze the TMB. In 2020 Yao et al. developed ecTMB to predict TMB using a statistical model to correct the panel design biases (Yao et al., 2020). Commercial solutions for the TMB calculation include Illumina (Illumina, 2021), ThermoFisher (Thermofisher, 2021), Qiagen (Qiagen, 2021), and Q2 Solutions (Q2solution, 2021). These solutions have been created specifically to calculate TMB in the clinical setting. However, their actual workflow is not known. The commercial tools (approved by FDA) recommended to establish the TMB are The FoundationOne CDx assay and the MSK-IMPACT (Memorial Sloan Kettering Cancer Center) panel, which have been authorized by the 510k pathway (Food U and Administration D, 2017b; Food U and Administration D, 2017a; Klempner et al., 2020).

Concerning cancer types, Melanoma has the highest mutation rate and the highest number of neoantigens (Snyder et al., 2014). Therefore, immunotherapy represents a common choice for its treatment. However, high-impact cancer disease on the lungs (Rizvi et al., 2015), prostate, colorectal (Stein et al., 2018), and breast could also benefit from this therapy. Monoclonal antibodies have shown promising efficacy against programmed death 1 (PD-1), such as Pembrolizumab for gastrointestinal (GI)-related cancer (Doi et al., 2018), or Nivolumab a PD-1 inhibitor used for patients with hepatocellular carcinoma (El-Khoueiry et al., 2017). Some monoclonal antibodies have already been used in angiogenesis for CRC, and there are two approved immune checkpoint inhibitors targeting PD-1 (pembrolizumab and nivolumab) used after progressed chemotherapy. In Cercek et al. (2022), authors show that 12 patients with advanced rectal cancer treated with single-agent PD-1 blockade had a complete clinical response, with no evidence of tumor on magnetic resonance imaging.

The gold standard to measure the TMB is the Whole Exome Sequencing (WES) analysis with tumor and normal samples. WES analysis, however, has a high cost and requires extensive data management. Indeed, two samples are needed to discard germline mutations. Unfortunately, the availability of these matched samples in clinical practice varies across organizations. Germline variants in tumor-only sequencing can be filtered out using available databases. Still, this procedure needs a high level of standardization for each type of tumor and each population (Meléndez et al., 2018). Therefore, to go beyond such a limitation, targeted panels are under investigation (Campesato et al., 2015; Johnson et al., 2016) to speed up the analysis and keep high precision and sensitivity. Some authors recommend using targeted gene panels, ideally with 1 Megabase as the lower bound limit to yield reliable TMB estimation (Allgäuer et al., 2018; Buchhalter et al., 2018; Endris et al., 2018).

Furthermore, differential expression genes (DEGs) analysis has clarified genes’ role in cancer patients between high and low TMB patients. Comparing tumor and normal colon samples, Gao et al. (2018) found that DEGs were mainly involved in protein transport, apoptotic, and neurotrophin signaling pathways. Wang et al. (2020) screened the TCGA-BRCA dataset splitting patients in TMB high and TMB low and analyzed them with the Kyoto Encyclopedia of Genes and Genomes (KEGG) and Gene Ontology (GO) databases. They found that DEGs were primarily enriched in epidermis development, extracellular matrix, and receptor-ligand activity among Biological processes, Cellular Components, and Molecular Functions, respectively. Zhang et al. (2020) found that differential genes were involved in catalytic activity in bladder urothelial carcinoma, acting on DNA, single−stranded DNA−dependent ATPase activity. Moreover, TMB enrichment of related signatures correlated with multiple cancer-related crosstalks, including cell cycle, DNA replication, cellular senescence, and p53 signaling pathway.

This study presents a Docker-based pipeline designed for pan-cancer applications, specifically aimed at computing the Tumor Mutational Burden (TMB) in DNA-Seq samples. Leveraging data from The Cancer Genome Atlas (TCGA), our research delves into the prospect of stratifying patients’ TMB using different approaches with the aim of identifying a small pan-cancer mutational signature allowing to effectively predict the actual TMB. Through our computational model, we identified a small signature composed of 389 genes showing a strong differential mutation rate between high and low TMB patients. Our findings reveal that such a signature effectively stratifies patients without necessitating Whole Exome Sequencing (WES), thus establishing its suitability for clinical contexts. We have rigorously validated its reliability, employing multiple independent datasets from TCGA and the European Genome Archive (EGA).

To further analyze the implications of this gene signature, we conducted a functional analysis for both the High TMB (H-TMB) and Low TMB (L-TMB) groups. Remarkably, our results highlight either the perturbation of several immunity-related pathways and the enrichment of many immune-involved Transcription Factors, shedding light on the potential biological significance of our findings.

## 2 Materials and methods

The forthcoming sections present an in-depth description of our methodology, delineating the comprehensive pipeline that integrates both upstream and downstream analyses. Our entire approach was constructed upon the foundation of the TCGA Harmonized Cancer Dataset, and its efficacy was rigorously examined across several independent datasets sourced from the EGA.

### 2.1 Datasets

We downloaded raw Colon adenocarcinoma samples used as our study’s primary tumor. BAM cancer and normal tissue biopsy files were analyzed for each sample, extracting the somatic mutations. All analyses have been repeated for five thresholds. Other analyzed solid cancer types were: Ovarian serous cystadenocarcinoma (OV, *n* = 441); Cervical squamous cell carcinoma and endocervical adenocarcinoma (CESC, *n* = 305); Thyroid carcinoma (THCA, *n* = 496); Bladder Urothelial Carcinoma (BLCA, *n* = 412); Uterine Corpus Endometrial Carcinoma and Uterine Carcinosarcoma (UCEC and UCS, *n* = 628); Esophageal carcinoma (ESCA, *n* = 181); Kidney renal papillary cell carcinoma (KIRP, *n* = 288); Kidney renal clear cell carcinoma (KIRC, *n* = 339); Liver hepatocellular carcinoma (LIHC, *n* = 415); Stomach adenocarcinoma (STAD, *n* = 450); Pancreatic adenocarcinoma (PAAD, *n* = 183); Prostate adenocarcinoma (PRAD, *n* = 497); Adrenocortical carcinoma (ACC, *n* = 240); Skin Cutaneous Melanoma (SKCM, *n* = 466); Lung Squamous Cell Carcinoma (LUSC, *n* = 494); Lung Adenocarcinoma (LUAD, *n* = 512). For these cancers, we directly downloaded the VCF files supplied by TCGA.

To test our signature we employed six independent datasets. These comprise 126 Colon Cancer WES obtained from TCGA; 72 colon WES samples from Genentech (Seshagiri et al., 2012); 42 samples taken from Colonomics (Díez-Villanueva et al., 2022); 101 Breast Cancer WES samples obtained from dbGaP (NCBI, 2021); 291 colon samples from COCA-CN study taken from ICGC (Zhang et al., 2019) and 338 samples from LINC-JP taken from ICGC. The first three datasets were analyzed with our pipeline starting from the BAM files. While, the somatic mutation files of the latter two, from ICGC, have been directly annotated using Annovar.

### 2.2 The upstream pipeline

**FIGURE 1:**
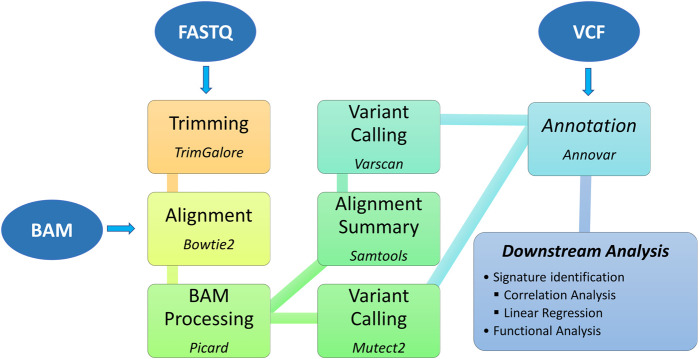
Bioinformatics pipeline: upstream and downstream analysis.

The TMBCalc pipeline (see Figure 1) has been implemented in Bash and R. It computes the TMB, yielding as output (i) the TMB value and (ii) a list of the mutations implicated in its calculation. The source code and user manual are available at https://github.com//knowmics-lab/TMBCalc. In addition, the containerized software can be downloaded with Docker through the DockerHub Container Image Library tmbcalc.

Our pipeline comprises five modules:

1. Alignment: WES BAM files are converted in FASTQ with the “bam2fq” command [Samtools (Li et al., 2009)]. Next, FASTQ files are aligned with Bowtie 2 (Langmead and Salzberg, 2012) using the hg38 Genome assembly gathered from NCBI.
2. BAM Processing: First, each BAM file is modified by adding a read group to each sequencing read using the “AddOrReplaceReadGroups” command in Picard tool (Picard toolkit, 2018). Then, all reads are sorted by genomic coordinate [“SortSam” command (Picard toolkit, 2018)] and contig ordering [“ReorderSam” command (Picard toolkit, 2018)]. Finally, duplicates are identified and removed [“MarkDuplicates” command (Picard toolkit, 2018)].
3. Variant calling: Gatk (v4.2.5.0) Mutect2 (McKenna et al., 2010) is employed to perform variant calling. In this step, all mutations labeled as “PASS” were retained. Furthermore, we run a second variant calling using the “somatic” and “somaticFilter” commands of VarScan (v2.4.4) (Koboldt et al., 2012) (min-var-freq = 10). This dual-phase procedure allows us to get a more precise variant calling by intersecting the two VCF output files.
4. Annotation: In this step, we remove all variants known to be unusable for computing TMB (Chalmers et al., 2017). Therefore, first, we annotate all inferred mutations with Annovar (Wang et al., 2010; Yang and Wang, 2015). The following databases are used: 1000genome (2015_08) (Consortium et al., 2015), snp146 (Sherry et al., 2001), cosmic88 (Tate et al., 2018), NHLBI Exome Sequencing Project (ESP6500) (Nhlbi go exome sequencing project, 2021). Then, variants that present annotations in these databases are filtered out, yielding a file containing only mutations valid for the TMB computation.
5. TMB calculation: This step produces two outputs: (i) the TMB value and (ii) the list of contributing variants. Although the TMB is commonly defined as “the number of the counted non-synonymous mutations that alter the amino acid sequence of a protein,” we also decided to include synonymous ones, following (Chalmers et al., 2017; Klempner et al., 2020), since this inclusion might improve sensitivity. Furthermore, such mutations are indicative of a mutational process. Therefore, the TMB has been computed as

$$TMB=\frac{\#mutations}{GenomeSize}$$ (1)

where *#mutations* is the number of mutations identified in the sample, and the genome size is 38 MB (Chalmers et al., 2017).

Given the absence of well-defined TMB thresholds, our analyses incorporated numerous values documented in existing literature pertaining to colon cancer. The selected thresholds were as follows: 5 mutations per megabase (5mut/Mb), 10 mutations per megabase (10mut/Mb), 20 mutations per megabase (20mut/Mb) as referenced in (Chalmers et al., 2017) mutations per megabase (25.29mut/Mb) calculated using the formula $TMB+1.25\times IQR\left(TMB\right)$ as outlined in (Fernandez et al., 2019), where IQR is the Interquartile range, and 34.66 mutations per megabase (34.66mut/Mb) as cited in (Wu et al., 2019a).

### 2.3 The Downstream pipeline

To create a gene signature panel suitable for the TMB analysis, our study started from the “AmpliSeq for Illumina Comprehensive Cancer Panel” - a pre-built Illumina panel of length 1.7 Mb, that covers all exons of 409 cancer-associated genes. To derive the size of each gene we make use of the R package EDAseq (Risso et al., 2011) with the “getGeneLengthAndGCContent” function. We measured its discriminant power in identifying patients with high and low TMB.

Next, we investigated the predictive efficacy of custom panels by conducting two parallel analyses. Initially, we curated sets of randomly selected genes, varying in size (50, 100, 200, and 300 genes), featuring mutations from WES data obtained from colon cancer patients. This process was iterated 1,000 times for each panel size to ensure robustness. Subsequently, we replicated the analysis using the most commonly mutated genes observed in colon cancer WES data, thus constructing panels comprising 50, 100, 200, 300, 409, and 500 genes. The primary objective was to juxtapose panels derived from randomly selected genes against those enriched with frequently mutated ones, thereby facilitating a comparative assessment of their predictive capabilities.

Subsequently, we constructed a concise signature by carefully selecting the top 10 most frequently mutated genes across 17 distinct cancer types. This curation resulted in a signature comprising 44 genes (refer to Supplementary Table S1), encompassing a genomic length of 1.05 Mb.

Finally, we devised a panel comprising genes exhibiting a noteworthy discrepancy in mutational signatures between high and low TMB (Tumor Mutational Burden) patients. To achieve this objective, we employed the following methodology. Initially, for each cancer type, we calculated the mutation rate per kilobase (kb) of each gene *g*_*i*_ in each patient *P*_*j*_, denoted as $MutationRate\left(g_{i},P_{j}\right)$ , using the formula:

$$MutationRate\left(g_{i},P_{j}\right)=\frac{M\left(g_{i},P_{j}\right)}{length\left(g_{i}\right)}\times 1000$$ (2)

where $M\left(g_{i},P_{j}\right)$ is the number of mutations for gene *g*_*i*_ in patient *P*_*j*_ and $length\left(g_{i}\right)$ is the length of gene *g*_*i*_ in bases.

Subsequently, we compared the gene mutation rates between H-TMB and L-TMB patients against the expected rates. A significant deviation from the expected value suggested that such differences could not be solely attributed to gene length. To ascertain the statistical significance of such differences, we conducted Mann-Whitney tests and corrected the *p*-values for multiple hypotheses using the Benjamini–Hochberg False Discovery Rate (FDR) method. Then, for each statistically significant gene *g*_*i*_, we computed its rank as the difference between the expected gene mutation rates in H-TMB and L-TMB:

$$rank\left(g_{i}\right)=E_{H-TMB}\left[MutationRate\left(g_{i},P_{j}\right)\right]-E_{L-TMB}\left[MutationRate\left(g_{i},P_{j}\right)\right]$$ (3)

Finally, we aggregated the rankings of all genes (one from each cancer type) and selected the top *k* genes to approximate a total size of 1 Mb (1.08 Mb). This methodology enabled us to construct a signature composed of 389 genes, facilitating robust analysis and inference.

## 3 Results

For each panel, we conducted a comprehensive data analysis involving the computation of correlation and logistic regression. In particular, we started annotating the mutated genes in all samples using the “RefSeq gene” Annovar database (Pruitt et al., 2012); after that, we correlated, using Pearson, the TMB calculated using WES with the one calculated using our custom gene signatures and the “AmpliSeq for Illumina Comprehensive Cancer Panel”; finally, we classified the patients using the R package caret (caret, 2016) employing the 10-fold cross-validation procedure to measure gene signatures reliability and calculating measures such as sensitivity (TPR), specificity (TNR), Positive Predictive Value (PPV), Negative Predictive Value (NPV).

### 3.1 Mutational Burden on custom gene panels

We conducted a comprehensive analysis of custom gene panels to compute the Tumor Mutational Burden (TMB), comparing their performance with whole-exome sequencing (WES) data. As anticipated, increasing the number of genes in the signature naturally boosts the correlation. Notably, even a modest set of 50 randomly selected genes exhibits a strong correlation with WES-derived TMB (refer to the Correlation column of Table 1). Upon stratifying patients into High-TMB (H-TMB) and Low-TMB (L-TMB) groups using a threshold of 20 mutations per megabase (mut/Mb), the correlation notably diminishes within the L-TBM class (see Table 1). The 500-gene panel maintains a robust correlation within the L-TMB class, albeit its larger size of 6.44 Mb justifies this correlation. Remarkably, our results reveal that a 389-gene signature maintains a high correlation within the L-TMB class despite its smaller size of 1.08 Mb (refer to Supplementary Tables S1, S2 for additional threshold analyses).

**TABLE 1:** For each random group of panels, we have calculated the average values of the follwing quantities: panel length, correlation, precision, recall, specificity and negative predicted value. For other gene panels,we have calculated their correlation, precision, recall, sensitivity and negative predicted value. The threshold used was 20mut/Mb. The table also reports the confusion matrix of each experiment.

Number of genes and panel length		Pearson correlation	H-TMB correlation	L-TMB correlation	PPV	TPR	TNR	NPV	TP	FP	TN	FN
50	*0.27* * * *R*	0.94	0.90	0.31	0.95	0.79	0.96	0.95	9.4	**0.5**	46.5	**2.5**
	*1.08* * * *H*	0.97	0.95	0.81	**1**	**1**	**1**	**1**	**12**	0	**47**	0
100	*0.55* * * *R*	0.97	0.94	0.42	0.98	0.88	0.99	0.97	10.63	0.23	46.77	1.37
	*1.80* * * *H*	0.98	0.97	0.86	**1**	**1**	**1**	**1**	**12**	0	**47**	0
200	*1.09* * * *R*	0.98	0.97	0.54	0.99	0.94	**1**	0.98	11.27	0.07	46.93	0.73
	*3.05* * * *H*	0.98	0.97	0.88	**1**	**1**	**1**	**1**	**12**	0	**47**	0
300	*1.64* * * *R*	**0.99**	**0.98**	0.62	**1**	0.95	**1**	0.99	11.44	0.02	46.98	0.56
	*4.28* * * *H*	**0.99**	**0.98**	0.90	0.91	**1**	**1**	**1**	**12**	0	**47**	0
409	*5.49* * * *H*	**0.99**	**0.98**	0.92	**1**	**1**	**1**	**1**	**12**	0	**47**	0
	*1.7 A*	0.93	0.88	0.56	**1**	**1**	**1**	**1**	**12**	0	**47**	0
500	*6.44* * * *H*	**0.99**	**0.98**	**0.93**	**1**	**1**	**1**	**1**	**12**	0	**47**	0
44	*1.05 C*	0.97	0.96	0.74	**1**	0.83	**1**	0.96	10	0	**47**	2
389	*1.08 C*	0.97	0.96	0.82	**1**	**1**	**1**	**1**	**12**	0	**47**	0

The bold values in Table 1 refer to the highest values.

Furthermore, we investigated the predictive capability of these panels. We categorized the actual TMB derived from WES into two classes, H-TMB and L-TMB, based on a 20 mut/Mb threshold (see Supplementary Material for alternative thresholds). This categorization was then utilized for logistic regression analysis. Table 1 presents the classification outcomes, demonstrating that TMB calculated with the gene panels effectively aligns with WES data. Particularly noteworthy is the performance of the 389-gene signature, which outperforms others when normalized by signature length.

### 3.2 *In-silico* 389 genes signature validation

**FIGURE 2:**
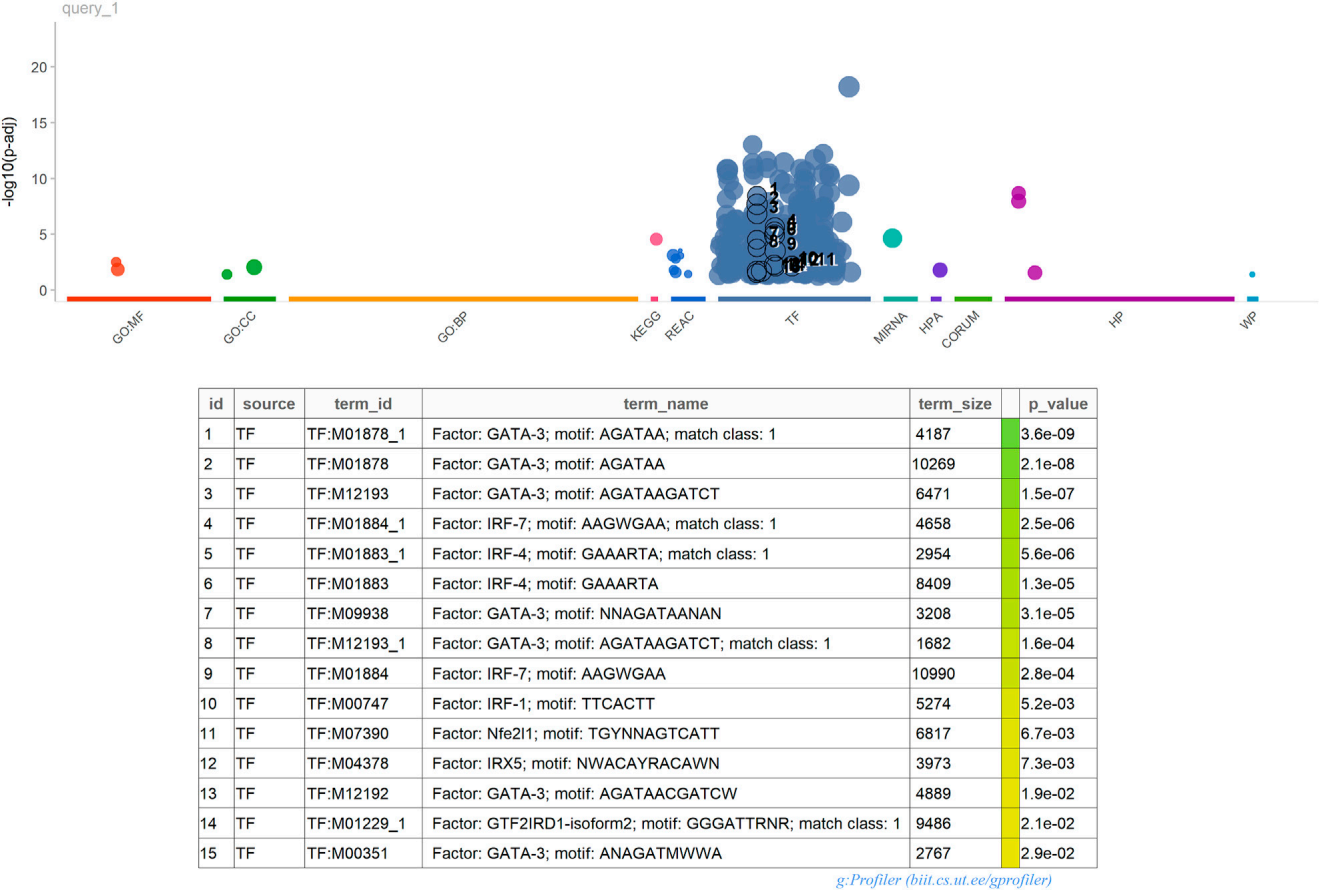
gProfiler2 Functional Analysis.

We extended our analysis to the entire TCGA cancer dataset, focusing on the 389 genes signature panel. As depicted in Table 2, the Pearson correlation across all tumors exceeds 85%. Notably, the correlation within the High-TMB (H-TMB) and Low-TMB (L-TMB) groups surpasses that of the 44-gene panel (see Supplementary Material). Additionally, employing logistic regression allowed us to establish precision and recall metrics. Across all tumors, we observed minimal occurrences of False Positives and False Negatives, with a consistently high Specificity exceeding 0.90 Figure 2.

**TABLE 2:** Correlation, Precision, and Recall of each TCGA tumor between TMB analyzed with the panel built with the 389 and WES TMB using threshold 20 mut/Mb.

	Pearson	Pearson H-TMB	Pearson L-TMB	PPV	TPR	TNR	NPV	TP	FP	TN	FN
UCEC-UCS	0.98	0.98	0.80	1.00	0.93	1	0.95	42	0	62	3
STAD	0.97	0.96	0.84	0.82	0.87	0.95	0.97	14	3	63	2
SKCM	0.98	0.98	0.86	0.91	0.86	0.90	0.95	44	4	37	7
BLCA	0.95	0.96	0.85	1.00	0.78	1	0.97	7	0	72	2
CESC	0.99	0.99	0.80	1	1	1	1	8	0	51	0
LUSC	0.93	0.91	0.82	0.78	0.5	0.96	0.86	11	3	73	11
LUAD	0.97	0.91	0.82	0.77	0.96	0.91	0.98	23	7	70	1
LIHC	0.93	0.86	0.83	0.5	0.50	0.99	0.99	1	1	76	1
THCA	0.99	1	0.86	0.67	1	0.99	1	2	1	95	0
OV	0.91	0.86	0.83	0.75	0.43	0.97	0.90	3	1	35	4
ESCA	0.90	0.92	0.82	-	0.75	1	0.88	0	0	32	4
PRAD	0.99	1	0.67	-	0	1	0.99	0	0	97	1
PAAD	0.99	1	0.76	1	1	1	1	1	0	34	0
KIRC	0.99	1	0.70	-	0	1	0.98	0	0	67	1
KIRP	0.87	0.97	0.81	-	0	1	0.98	0	0	56	1
ACC	0.98	1	0.88	1	1	1	1	1	0	43	0

The table also reports the True Negative Rate, Negative Predicted Value, and the confusion matrix for each cancer type.

We also validated the panel’s performance across six independent datasets (see Table 3):

•   126 TCGA COAD samples that were not utilized in creating the signature. Their correlation stands at 0.95 for H-TMB and 0.85 for L-TMB.
•   72 samples from Genentech exhibit a high level of correlation between TMB calculated with WES and the signature gene panels. This correlation is 0.95 for the H-TMB patients and 0.87 for the L-TMB ones.
•   291 COCA-CN colon cancer samples from EGA demonstrate an excellent correlation between TMB calculated on the WES and the gene panels (0.99). This correlation persists within the H-TMB group, while the L-TMB group has a correlation coefficient equal to 0.8.
•   42 colon cancer samples from Colonomics show a Pearson correlation of 0.69. However, since all patients have Low TMB, we could not determine a correlation for the H-TMB group.
•   dbGaP, consisting of 101 breast cancer samples, shows a Pearson correlation of 0.89. Upon splitting the samples into H-TMB and L-TMB with a threshold of 10 mut/Mb, the panel exhibits a correlation of 1 for H-TMB and 0.86 for L-TMB.
•   338 Liver-JP samples from ICGC show that although the H-TMB correlation coefficient is as high as in colon cancer, the L-TMB decreases to 0.73. Despite this decrease, the correlation level can still be considered moderate.

**TABLE 3:** Correlation, precision, and recall between TMB computed with WES and 389 genes panel using thresholds 20mut/Mb for each cancer except dbGaP where the threshold was 10 mut/Mb.

	Pearson correlation	Pearson H-TMB	Pearson L-TMB	PPV	TPR	TNR	NPV	TP	FP	TN	FN
Genentech	0.94	0.95	0.87	1	1	1	1	2	0	11	0
Colonomics	0.69	-	0.69	-	-	-	-	-	-	8	-
Li-JP	0.99	0.99	0.73	0.87	0.87	0.68	0.68	42	6	13	6
TCGA 126 COAD	0.97	0.95	0.85	0.75	1	0.95	1	3	1	20	0
dbGap*	0.89	1	0.86	-	0	1	0.95	0	0	18	1
COCA	0.99	0.99	0.80	0.89	0.62	1	0.94	5	0	50	3

The table also reports the True Negative Rate, Negative Predicted Value, and the confusion matrix for each cancer type.

Therefore, we can conclude that the 389 gene panel is a promising solution to assess the TMB with reasonable accuracy for many cancer types. Furthermore, due to the relatively small size, it could speed up the TMB analysis, cutting the costs while reaching in principle the same results as WES (see Supplementary Table S5 for the same analysis on the 44 genes panel).

### 3.3 Signature functional analysis

We conducted a comprehensive functional analysis using the 389-gene signature. Differential Expression Analysis (DEGs) was performed on TCGA RNA-seq data by using the raw counts (HTSeq-Counts) for the 389 genes of the panel. We collected the expression data with TCGAbiolinks (Colaprico et al., 2015; Silva et al., 2016; Mounir et al., 2019) and performed the analysis with Limma (Ritchie et al., 2015). Genes were considered differentially expressed if $\left|log_{2}FC\right|\leq log_{2}\left(1.5\right)$ and adjusted *p*-value <0.05. Pathway perturbation and enrichment analysis were conducted using MITHrIL (Alaimo et al., 2016; Alaimo et al., 2017). Additionally, gProfiler2 (Kolberg et al., 2020) was employed for gene set enrichment analysis.

**FIGURE 3:**
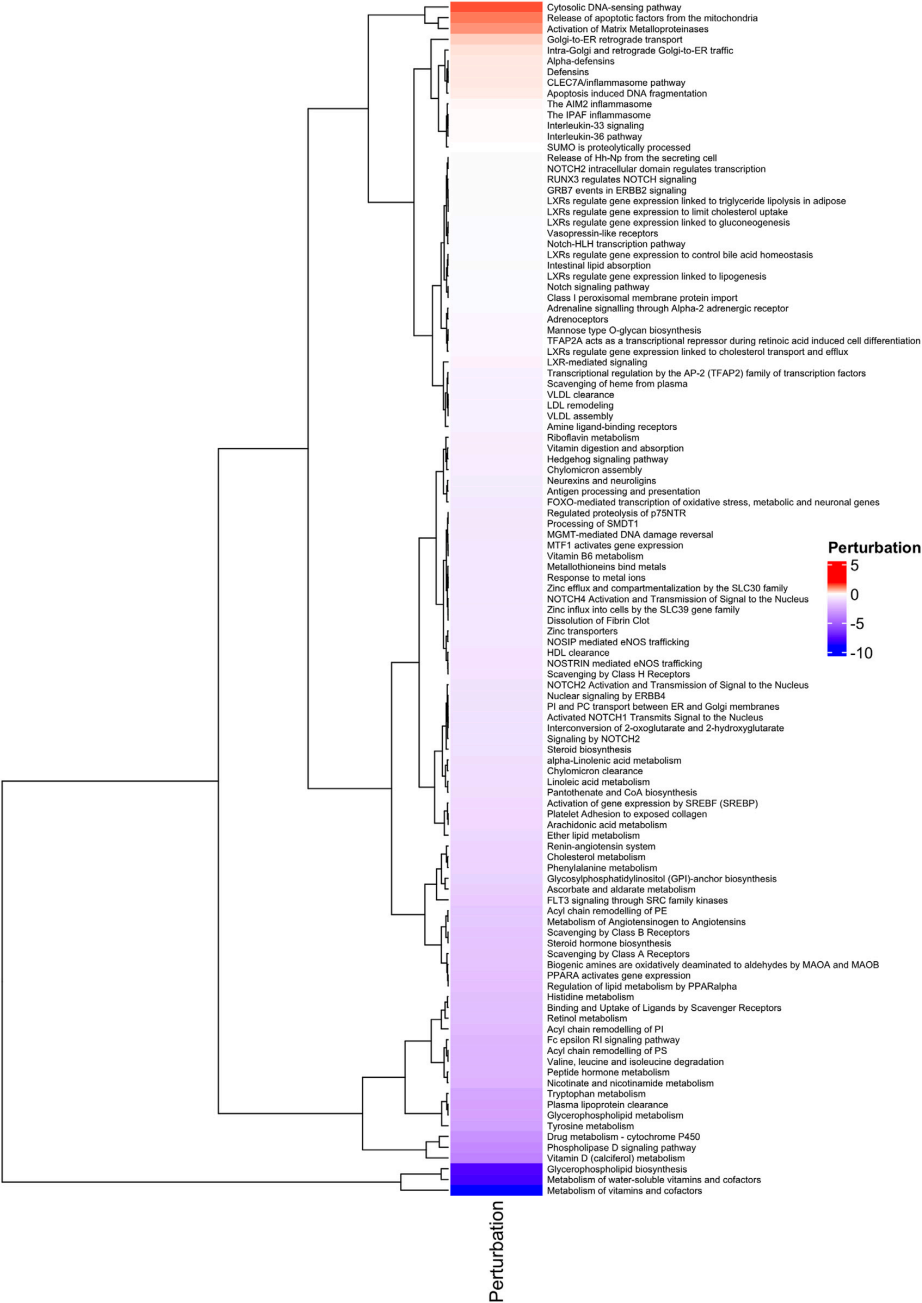
Functional Analysis. Heatmap of Perturbated Pathways with *p* ≤ 0.05, analyzed with MITHrIL in the 389 genes signature.

The comparative analysis between H-TMB and L-TMB patients across 17 tumors helped to elucidate differences in enriched pathways. Our investigation yielded significant outcomes emphasizing pathways intricately linked with immune and inflammatory responses. As depicted in Figure 3, we show substantial immune pathways, including (i) the Cytosolic DNA-sensing pathway, which exhibited upregulation in H-TMB patients, indicating innate immunity activation and serving as a potential adjuvant for anticancer immune therapy (Yu and Liu, 2021); and (ii) the defensins pathway, also upregulated, known for its role as immunomodulators and attractors of immune cells (Contreras et al., 2020).

Furthermore, enrichment analysis conducted using gProfiler2 3 highlighted a significant presence of Transcription Factors (TFs) within our 389 gene signature. This enrichment suggests that these TFs likely play pivotal roles in regulating the expression of these genes. Understanding the impact of these TFs on the immune system holds crucial implications. For instance, NF-*κ*B, AP-1, IRF, and GATA3, prominent regulators of immune responses, govern the expression of genes involved in inflammation, cytokine production, cell differentiation, and immune cell activation. By comprehending the regulatory mechanisms of these TFs on immune-related genes, valuable insights can be gained into immune responses against pathogens, antigens, and environmental stimuli. For example, GATA3 is critical in differentiating T helper 2 (Th2) cells, which produce cytokines crucial for allergic responses and immune regulation. Moreover, the interplay between Transcription Factors, including NF-*κ*B, AP-1, IRF, and GATA3, often involves intricate interactions with each other and with other TFs to orchestrate coordinated immune responses.

## 4 Discussion

The immune response holds considerable promise in combating tumors. However, as tumors progress, they evolve mechanisms to evade detection by the immune system, often by producing a range of inhibitory molecules. Immunotherapy, leveraging immune checkpoint inhibitors, works to remove these brakes on the immune response. Tumors harboring a higher load of neoantigens and mutations tend to derive greater benefit from this approach, as they can provoke a more robust immune response. Identifying patients who are suitable candidates for immunotherapy relies heavily on Tumor Mutational Burden (TMB) assessment. The computation and analysis of TMB necessitate sophisticated Next-Generation Sequencing (NGS) techniques and advanced bioinformatics expertise. Efforts within the scientific community are directed towards streamlining this process for clinical application. By obtaining a molecular portrait of the tumor, precision medicine approaches in immunotherapy can be realized.

Currently, Whole Exome Sequencing (WES) remains the preferred method for TMB computation, although there are several panel-based alternatives available to expedite the analysis. Notably, two such panels have already received FDA approval. We have highlighted the utility of the AmpliSeq for Illumina Comprehensive Cancer Panel as a novel tool for TMB analysis. This panel, commonly utilized in laboratories for identifying variants across various cancer types, offers the advantage of incorporating additional markers for cost-effective TMB evaluation. This parallels the trend seen in some NGS panels, which now include assessment markers for Microsatellite Instability (MSI). While cancer-specific panels may offer improved accuracy for TMB estimation, a unified panel for multiple analyses presents a more cost-effective and flexible solution for laboratories dealing with diverse tumor types. Wu et al. (2019b) note that currently available Next-Generation Sequencing (NGS) panels can accurately assess Tumor Mutational Burden (TMB) only within specific cancer types. They emphasize the importance of relying on accuracy rather than correlation when evaluating panel performance. Notably, existing TMB commercial panels have been evaluated solely based on correlation, achieving coefficients of 0.74 for the FDA-approved FoundationOne CDx Panel and 0.76 for the MSK-IMPACT panel (Chalmers et al., 2017; Zehir et al., 2017; Wu et al., 2019b).

Our analysis emphasizes an alternative method for stratifying patients for immunotherapy by utilizing our 389 gene panel. Our panel, assessed across 17 tumor types, exhibits higher correlation coefficients (refer to Table 2 and Supplementary Table S4) and demonstrates favorable outcomes in regression analysis measured by Precision/Recall. The genesis of our 44 gene panel stemmed from identifying the top-10 most mutated genes in colon cancer. These genes, consistently highlighted in various studies as highly mutated (Kang et al., 2020a), have been subject to extensive functional investigations. For instance, Jia et al. (2019) established that mutations in *TTN* predict elevated TMB and increased response rates to Immune checkpoint blockade immunotherapy. Additionally, they observed favorable clinical outcomes correlated with *TTN* mutations. Furthermore, within the 389 gene signature panel, we identified genes such as (i) ACE2, recognized for its involvement in innate immunity; (ii) TRIM51, which exhibits increased expression in patients with high immune cell infiltration (Chen et al., 2022); (iii) SERPINB4, whose high mutation frequency correlates with improved survival post-immunotherapy in melanoma patients (Riaz et al., 2016); and (iv) ADAM2, whose expression enhances the cytotoxicity of CD8 T-cells (Dervovic et al., 2023). These findings underscore the potential of our panel not only for TMB assessment but also for uncovering crucial immunoregulatory mechanisms underlying tumor response to therapy.

Building upon the insights of Wang et al. (2020) and Kang et al. (2020b), our analysis delved into the identification of Differentially Expressed Genes (DEGs) between the two Tumor Mutational Burden (TMB) groups, elucidating their impact on signaling pathways. Our findings revealed perturbations in numerous immunity pathways, highlighting the significance of our signature in modulating immune and inflammatory responses. Consequently, mutations in these genes or alterations in their activity are likely to exert a substantial influence on immune responses, thereby affecting the efficacy of immunotherapy interventions.

## 5 Limitations of the study

The main limitation of our study is the lack of patients who have undergone immunotherapy to understand better our panel role in predicting its clinical outcome, in a future work we aim to investigate those data. Moreover, the lack of standardization for the TMB thresholds makes determining the best one for each cancer type impossible. Indeed, clinical trials are needed to confirm our data.

## Data Availability

Publicly available datasets were analyzed in this study. This data can be found here: https://www.cancer.gov/tcga, https://www.ncbi.nlm.nih.gov/gap/, https://dcc.icgc.org/.
