# Supplementary material for: TMBcalc: a computational pipeline for identifying pan-cancer Tumor Mutational Burden gene signatures
Source: Front Genet. 2024 Apr 5;15:1285305. doi: 10.3389/fgene.2024.1285305 (PMC11026579; doi:10.3389/fgene.2024.1285305)
Supplement: Supplementary file 2 [file DataSheet1.pdf]

Supplementary Material

Number of Genes (Panel mean length)	Threshold mut/Mb	Pearson	Pearson High TMB	Pearson Low TMB	PPV	TPV	TNR	NPV	TP	FP	TN	FN
50 (0.27)	5	0.94	0.93	0.17	0.83	0.47	0.91	0.70	11.9	2.9	31.3	13.1
	10		0.90	0.25	0.94	0.69	0.98	0.96	11.1	0.78	45.2	1.93
	20		0.90	0.31	0.95	0.79	0.96	0.95	9.4	0.5	46.5	2.5
	25.29		0.89	0.36	0.93	0.87	0.98	0.97	9.5	0.8	47.1	1.4
	34.66		0.89	0.56	0.88	0.84	0.97	0.97	8.4	1.2	47.8	1.5
100(0.55)	5	0.97	0.96	0.24	0.86	0.52	0.93	0.73	13.04	2.2	31.78	11.96
	10		0.95	0.34	0.97	0.92	0.99	0.98	11.91	0.39	45.61	1.09
	20		0.94	0.42	0.98	0.88	0.97	0.97	10.63	0.23	46.77	1.37
	25.29		0.94	0.49	0.95	0.85	0.99	0.99	10.49	0.64	47.36	0.5
	34.66		0.94	0.69	0.91	0.92	0.98	0.98	9.2	1.01	47.99	0.78
200(1.09)	5	0.98	0.98	0.33	0.88	0.56	0.94	0.75	14.1	2.1	31.9	10.9
	10		0.97	0.45	0.98	0.96	0.99	0.99	12.53	0.2	45.78	0.5
	20		0.97	0.54	0.99	0.94	1	0.98	11.27	0.07	46.93	0.73
	25.29		0.97	0.62	0.96	0.99	0.99	1	10.88	0.52	47.48	0.12
	34.66		0.97	0.80	0.93	0.96	0.98	0.99	9.61	0.73	48.27	0.39
300(1.64)	5	0.99	0.99	0.39	0.88	0.59	0.94	0.76	4.85	2.08	31.92	10.15
	10		0.98	0.53	0.99	0.98	0.99	0.99	12.79	0.18	45.81	0.2
	20		0.98	0.62	1	0.95	1	0.99	11.44	0.02	46.98	0.56
	25.29		0.98	0.69	0.96	0.99	0.99	1	10.94	0.49	47.51	0.06
	34.66		0.98	0.86	0.95	0.97	0.99	0.99	9.74	0.57	48.43	0.25

Table S1. Table of correlation between WES TMB and TMB computed using random mutated genes panels of different sizes. For each group of panels, we have calculated the mean of correlation, PPV, TPR, TNR and NPV. The analysis have been performed for each threshold considered in the paper.

**Number of Genes (Panel mean length)**	**Threshold mut/Mb**	**Pearson**	**Pearson High TMB**	**Pearson Low TMB**	**PPV**	**TPR**	**TNR**	**NPV**	**TP**	**FP**	**TN**	**FN**
500 (6.44)	**5**	0.99	0.99	0.77	0.95	0.76	0.97	0.85	19	1	33	6
	**10**		0.98	0.89	1	1	1	1	13	0	46	0
	**20**		0.98	0.93	1	1	1	1	12	0	47	0
	**25.29**		0.98	0.95	0.92	1	0.98	1	11	1	47	0
	**34.66**		0.98	0.97	1	1	1	1	10	0	49	0
409 (5.49)	**5**	0.99	0.99	0.74	0.91	0.8	0.94	0.86	20	2	32	5
	**10**		0.98	0.88	1	1	1	1	13	0	46	0
	**20**		0.98	0.92	1	1	1	1	12	0	47	0
	**25.29**		0.98	0.94	0.92	1	0.98	1	11	1	47	0
	**34.66**		0.98	0.96	1	1	1	1	10	0	49	0
300 (4.28)	**5**	0.99	0.98	0.70	0.91	0.80	0.94	0.86	20	2	32	5
	**10**		0.98	0.85	1	1	1	1	13	0	46	0
	**20**		0.98	0.90	1	1	1	1	12	0	47	0
	**25.29**		0.98	0.93	0.92	1	0.98	1	11	1	47	0
	**34.66**		0.98	0.96	0.91	1	0.98	1	10	1	48	0
200 (3.05)	**5**	0.98	0.98	0.65	0.86	0.76	0.91	0.84	19	3	31	6
	**10**		0.97	0.82	1	1	1	1	12	0	47	0
	**20**		0.97	0.88	1	1	1	1	12	0	47	0
	**25.29**		0.97	0.91	0.92	1	0.98	1	11	1	47	0
	**34.66**		0.97	0.94	0.91	1	0.98	1	10	1	48	0
100 (1.8)	**5**	0.98	0.97	0.60	0.87	0.80	0.91	0.86	20	3	31	5
	**10**		0.97	0.79	1	1	1	1	13	0	46	0
	**20**		0.97	0.86	1	1	1	1	12	0	47	0
	**25.29**		0.97	0.89	0.92	1	0.98	1	11	1	47	0
	**34.6**		0.96	0.92	0.91	1	0.98	1	10	1	48	0
50 (1.08)	**5**	0.97	0.96	0.55	1	0.76	1	0.85	19	0	34	6
	**10**		0.95	0.73	1	1	1	1	13	0	46	0
	**20**		0.95	0.81	1	1	1	1	12	0	47	0
	**25.29**		0.95	0.86	0.92	1	0.98	1	11	1	47	0
	**34.66**		0.95	0.88	0.91	1	0.98	1	10	1	48	0

Table S2. Most Frequently mutated genes panels correlation. Patients number: Threshold 5 mut/Mb 126 H-TMB, 172 L-TMB; Threshold 10 mut/Mb 66 H-TMB, 232 L-TMB; Threshold 20 mut/Mb 60 H-TMB, 238 L-TMB; Threshold 25.29 mut/Mb 58 H-TMB, 240 L-TMB; Threshold 34.66 mut/Mb 51 H-TMB, 247 L-TMB. PPV, TPR, TNR and NPV of all colon cancer thresholds have been calculated with logistic regression for all panels with the most mutated genes.

**Threshold mut/Mb**	**Pearson**	**Pearson High TMB**	**Pearson Low TMB**	**PPV**	**TPR**	**TNR**	**NPV**	**TP**	**FP**	**TN**	**FN**
**4.46**	0.93	0.92	0.40	0.86	0.62	0.90	0.70	18	3	26	11
**5**		0.92	0.42	0.87	0.52	0.94	0.73	13	2	32	12
**10**		0.89	0.41	0.93	1	0.98	1	13	1	45	0
**20**		0.88	0.56	1	1	1	1	12	0	47	0
**20.34**		0.88	0.56	1	1	1	1	12	0	47	0
**25.29**		0.87	0.62	0.91	0.91	0.98	0.98	10	1	47	1
**34.66**		0.86	0.75	0.90	0.90	0.98	0.98	9	1	48	1

Table S3. Correlation between WES and AmpliSeq for Illumina Comprehensive Cancer Panel. PPV, TPR, TNR and NPV of all colon cancer thresholds have been calculated with logistic regression for AmpliSeq for Illumina Comprehensive Cancer Panel

	**Pearson**	**Pearson H- TMB**	**Pearson L-TMB**	**PPV**	**TPR**	**TNR**	**NPV**	**TP**	**FP**	**TN**	**FN**
**UCEC-UCS**	0.98	0.98	0.77	1.00	0.93	1	0.95	42	0	62	3
**STAD**	0.97	0.97	0.79	0.88	0.94	0.97	0.98	15	2	64	1
**COAD**	0.97	0.96	0.74	1	0.83	1	0.96	10	0	46	2
**SKCM**	0.98	0.98	0.86	0.90	0.88	0.88	0.86	45	5	36	6
**BLCA**	0.93	0.95	0.74	1.00	0.78	1	0.97	7	0	72	2
**CESC**	0.99	0.99	0.70	0.87	0.87	0.98	0.98	7	1	50	1
**LUSC**	0.91	0.89	0.78	0.94	0.72	0.99	0.92	16	1	75	6
**LUAD**	0.95	0.90	0.89	0.74	0.83	0.91	0.94	20	7	70	4
**LIHC**	0.90	0.83	0.79	1.00	0.50	1	0.99	1	0	77	1
**THCA**	1	1	0.87	1.00	0.50	1	0.99	1	0	96	1
**OV**	0.92	0.88	0.80	0.62	0.71	0.92	0.94	5	3	33	2
**ESCA**	0.84	0.86	0.79	0.50	0.75	0.91	0.97	3	3	29	1
**PRAD**	0.99	1	0.55	NA	0	1	0.99	0	0	97	1
**PAAD**	1	1	0.65	1	1	1	1	1	0	33	0
**KIRC**	0.98	1	0.60	1	1	1	1	1	0	66	0
**KIRP**	0.83	0.98	0.74	0	0	0.96	0.98	0	2	54	1
**ACC**	0.94	0.93	0.81	NA	0	1	0.98	0	0	43	1

Table S4. Correlation, Precision, and Recall of each TCGA tumor between TMB analyzed with the panel built with the 44 most mutated genes and WES TMB using threshold 20 mut/Mb.The table also reports the True Negative Rate, Negative Predicted Value, and the confusion matrix for each cancer type.

1 SUPPLEMENTARY DATA

In Table. S3, we describe the results of the AmpliSeq for Illumina Comprehensive Cancer Panel. The correlation is very high without splitting the patients into H-TMB and L-TMB groups. However, while it stays stable for the H-TMB patients, the correlation strongly decreases for the L-TMB one. The comprehensive panel properly partitions the patients into High and Low TMB using logistic regression, showing high sensitivity and specificity. Therefore, such a panel could suit TMB computation in the clinical setting.

In what follows, we report the statistical analysis of each tumor.

	Threshold	Pearson	Pearson H-TMB	Pearson L-TMB	PPV	TPR	TNR	NPV	TP	FP	TN	FN
TCGA 126	5	0.96	0.94	0.57	1	0.6	1	0.90	3	0	19	2
	10		0.92	0.67	1	1	1	1	3	0	21	0
	20		0.91	0.77	0.75	1	0.95	1	3	1	20	0
	25.29		0.91	0.83	1	1	1	1	3	0	22	0
	34.66		0.89	0.94	1	1	1	1	2	0	23	0
Genentech	5	0.95	0.96	0.51	1	1	1	1	3	0	10	0
	10		0.96	0.55	1	1	1	1	3	0	11	0
	20		0.97	0.92	1	1	1	1	2	0	11	0
	25.29		0.97	0.92	NA	0	1	0.92	0	0	12	1
	34.66		0.99	0.95	NA	NA	1	1	0	0	13	0
COCA	5	0.97	0.97	0.65	0.84	0.67	0.91	0.79	2	0	23	0
	10		0.97	0.50	0.83	0.67	0.95	0.89	2	0	23	0
	20		0.97	0.64	1	0.50	1	0.92	4	0	50	4
	25.29		0.97	0.62	0.83	0.83	0.98	0.98	5	1	51	1
	34.66		0.97	0.72	1	0.6	1	0.96	3	0	53	2
Colonomics	5	0.72	0.35	0.47	1	0.33	1	0.71	1	0	5	2
	10		NA	0.72	1	1	NA	NA	NA	NA	NA	NA
	20		NA	0.72	NA	NA	NA	NA	NA	NA	8	NA
	25.29		NA	0.72	1	1	NA	NA	NA	NA	NA	NA
	34.66		NA	0.72	1	1	NA	NA	NA	NA	8	NA
Li-Jp	20	0.99	0.99	0.53	0.93	0.83	0.84	0.67	40	3	16	8
dbGap*	10	0.77	1	0.79	NA	0	1	0.95	0	0	18	1

Table S5. Correlation between WES TMB and 44 genes panel TMB using all colon cancer thresholds. PPV, TPR, TNR and NPV for the six datasets have been calculated trough logistic regression. All the colon thresholds have been used for Genentech, Colonomics, TCGA 126, and COCA. For Liver, we used only threshold 20 mut/Mb and threshold 10 mut/Mb for dbGaP. The table also reports the True Negative Rate, Negative Predicted Value, and the confusion matrix for each cancer type.

**Ten most frequently mutated genes**	**#mutation in all patients**	**Patients with the mutations**	**Mean mutations per patient (SD)**	**Patients with mutations and high TMB level (Out of 60)**	**Patients with mutations and low TMB level (Out of 238)**	**Mutations in H-TMB patients - Mean of mutations per high patient (SD)**		**Mutations in L-TMB patients - Mean mutations per low patient (SD)**		**Expected Mutation rate**	**Observed Mutation rate H-TMB**	**Observed Mutation rate L-TMB**
**TTN**	369	117 (39%)	3.15 (5.1)	55 (92%)	62 (26%)	298	5.42 (6.8)	71	1.14 (0.4)	0.010	0.042***	0.002***
**SYNE1**	301	109 (36%)	2.76 (3.5)	55 (92%)	54 (23%)	236	4.29 (4.4)	65	1.20 (0.5)	0.022	0.084***	0.005***
**MUC19**	214	91 (30%)	2.35 (2.7)	45 (75%)	46 (19%)	160	3.55 (3.4)	54	1.17 (0.5)	0.024	0.090***	0.008***
**RYR2**	209	92 (31%)	2.27 (2.9)	53 (88%)	39 (16%)	166	3.13 (3.6)	43	1.10 (0.3)	0.039	0.155***	0.010***
**NEB**	192	68 (23%)	2.82 (3.1)	46 (77%)	22 (9%)	166	3.61 (3.6)	26	1.18 (0.4)	0.021	0.090***	0.004***
**LRP1B**	169	88 (29%)	1.92 (1.9)	45 (75%)	43 (18%)	121	2.69 (2.4)	48	1.12 (0.3)	0.035	0.123***	0.012***
**MUC16**	168	75 (25%)	2.24 (2.9)	42 (70%)	33 (14%)	132	3.14 (3.7)	36	1.09 (0.3)	0.013	0.050***	0.003***
**DYNC2H1**	152	83 (28%)	1.83 (1.6)	52 (87%)	31 (13%)	119	2.89 (1.9)	33	1.06 (0.2)	0.034	0.132***	0.009***
**RYR3**	143	64 (21%)	2.23 (2.8)	40 (67%)	24 (10%)	116	2.9 (3.4)	27	1.12 (0.4)	0.024	0.095***	0.006***
**COL11A1**	141	74 (25%)	1.90 (2.2)	35 (58%)	39 (16%)	99	2.83 (3)	42	1.08 (0.3)	0.052	0.181***	0.019**

Table S6. Ten most frequently genes percentage, mean, standard deviation, and expected and observed mutation rate in Colon adenocarcinoma with threshold 20. The statistical significance: ***p-val$<$ 0.001, **p-val$<$ 0.01.The analysis clearly show that the mutation rate within the H-TMB class is much higher and the expected one. Conversely, the mutation rate within the L-TMB class is lower than the expected.

Ten most frequently mutated genes	Patients with mutations and H-TMB level (Out of 126)	Mutations in high TMB patients - Mean of mutations per high patient (SD)		Patients with mutations and low TMB level (Out of 172)	Mutations in low TMB patients - Mean of mutations per low patient (SD)	
**TTN**	82 (65%)	328	4 (5.9)	35 (20%)	41	1.17 (0.38)
**SYNE1**	74 (59%)	263	3.55 (4)	35 (20%)	38	1.08 (0.28)
**MUC19**	63 (50%)	180	2.86 (3.1)	28 (16%)	34	1.21 (0.56)
**RYR2**	70 (55%)	185	2.64 (3.3)	22 (13%)	24	1.09 (0.93)
**NEB**	55 (44%)	177	3.21 (3.4)	13 (7%)	15	1.15 (0.37)
**LRP1B**	63 (50%)	142	2.25 (2.2)	25 (14%)	27	1.08 (0.28)
**MUC16**	59 (47%)	152	2.58 (3.2)	16 (9%)	16	1 (0)
**DYNC2H1**	67 (53%)	134	2 (1.7)	16 (9%)	18	1.12 (0.35)
**RYR3**	52 (41%)	131	2.52 (3.05)	12 (7%)	12	1 (0)
**COL11A1**	51 (40%)	118	2.31 (2.6)	23 (13%)	23	1 (0)

Table S7. Ten most frequently genes percentage, mean, standard deviation, fisher and welch test in Colon adenocarcinoma with threshold 5 mut/Mb

Ten most frequently mutated genes	Patients with mutations and high TMB level (Out of 66)	Mutations in high TMB patients - Mean of mutations per high patient (SD)		Patients with mutations and low TMB level (Out of 232)	Mutations in low TMB patients - Mean of mutations per low patient (SD)	
**TTN**	58 (89%)	301	5.19 (6.7)	59 (25%)	68	1.15 (0.4)
**SYNE1**	57 (86%)	240	4.21 (4.4)	59 (25%)	61	1.03 (0.5)
**MUC19**	48 (73%)	164	3.42 (3.4)	43 (18%)	50	1.16 (0.5)
**RYR2**	56 (85%)	169	3.02 (3.6)	36 (18%)	40	1.11 (0.3)
**NEB**	46 (70%)	166	3.61 (3.6)	22 (9%)	26	1.18 (0.4)
**LRP1B**	49 (74%)	125	2.55 (2.4)	39 (17%)	44	1.13 (0.3)
**MUC16**	45 (68%)	135	3 (3.6)	30 (13%)	33	1.1 (0.3)
**DYNC2H1**	54 (82%)	121	2.24 (1.9)	29 (12%)	31	1.06 (0.3)
**RYR3**	41 (62%)	117	2.85 (3.3)	23 10%	26	1.13 (0.4)
**COL11A1**	40 (61%)	104	2.60 (2.9)	34 (15%)	37	1.09 (0.3)

Table S8. Ten most frequently genes percentage, mean, standard deviation, Fisher and Welch test in Colon adenocarcinoma with threshold 10 mut/Mb

Ten most frequently mutated genes	Patients with mutations and high TMB level (Out of 58)	Mutations in high TMB patients - Mean of mutations per high patient (SD)		Patients with mutations and low TMB level (Out of 240)	Mutations in low TMB patients - Mean of mutations per low patient (SD)	
**TTN**	53 (91%)	293	5.53 (6.9)	64 (27%)	76	1.19 (0.4)
**SYNE1**	54 (93%)	235	4.35 (4.5)	55 (23%)	66	1.20 (0.5)
**MUC19**	44 (76%)	158	3.59 (3.5)	47 (19%)	56	1.19 (0.5)
**RYR2**	51 88%	164	3.21 (3.7)	41 (17%)	45	1.10 (0.3)
**NEB**	46 79%	166	3.61 (3.6)	22 (9%)	26	1.18 (0.4)
**LRP1B**	45 75%	121	2.69 (2.4)	43 (18%)	48	1.17 (0.3)
**MUC16**	41 71%	131	3.19 (3.7)	34 (14%)	37	1.09 (0.3)
**DYNC2H1**	51 (88%)	118	2.31 (1.9)	32 (13%)	34	1.06 (0.2)
**RYR3**	39 (67%)	115	2.95 (3.4)	25 (10%)	28	1.12 (0.4)
**COL11A1**	33 (57%)	96	2.90 (3.1)	41 (17%)	45	1.10 (0.3)

Table S9. Ten most frequently genes percentage, mean, standard deviation, Fisher and Welch test in Colon adenocarcinoma with threshold 25.29 mut/Mb

**Ten most frequently mutated genes**	**Patients with mutations and high TMB level (Out of 51)**	**Mutations in high TMB patients - Mean of mutations per high patient (SD)**		**Patients with mutations and low TMB level (Out of 247)**	**Mutations in low TMB patients - Mean of mutations per low patient (SD)**	
**TTN**	49 (96%)	283	5.77 (7.1)	68 (27%)	86	1.26 (0.7)
**SYNE1**	49 (96%)	228	4.65 (4.6)	60 (24%)	73	1.22 (0.5)
**MUC19**	40 (78%)	153	3.82 (3.6)	51 (21%)	61	1.20 (0.5)
**RYR2**	46 (90%)	156	3.39 (3.8)	46 (19%)	53	1.15 (0.4)
**NEB**	44 (86%)	164	3.73 (3.6)	24 (10%)	28	1.17 (0.4)
**LRP1B**	41 (80%)	115	2.80 (2.5)	47 (19%)	54	1.15 (0.7)
**MUC16**	39 (76%)	129	3.31 (3.8)	36 (14%)	39	1.08 (0.3)
**DYNC2H1**	46 (90%)	111	2.41 (2)	37 (15%)	41	1.11 (0.3)
**RYR3**	37 (72%)	112	3.03 (3.5)	27 (11%)	31	1.15 (0.4)
**COL11A1**	31 (61%)	94	3.03 (3.2)	43 (17%)	47	1.09 (0.3)

Table S10. Ten most frequently genes percentage, mean, standard deviation, Fisher and Welch test in Colon adenocarcinoma with threshold 34.66 mut/Mb

**Tumor**	**Genes**
**COAD**	TTN – SYNE1 – MUC19 – RYR2 – NEB – LRP1B – MUC16 – DYNC2H1 – RYR3 –COL11A1
**ESCA**	TTN – MUC19 – LRP1B – CSMD3 – SNHG14 –SYNE1 – MUC16 – RYR2 – HMCN1 – COL11A1
**STAD**	TTN – SYNE1 – LRP1B – SNHG14 – CSMD3 – MYHAS – HMCN1 – MUC16 – DNAH5 – SPTA1
**LIHC**	ALB - TTN – CSMD3 – RYR2 – MUC19 – OBSCN – HMCN1 – COL11A1 – MUC16 – LRP1B
**PAAD**	TTN – SYNE1 – MUC16 – HMCN1 – LRP1B – SNHG14 – MACF1 – RYR3 – USH2A – NEB
**BLCA**	TTN – SNHG14 –SYNE1 – CSMD3 – HMCN1 – MYHAS –MUC16 –RYR2 –LRP1B –SYNE2
**KIRP**	TTN – LRP2 –SYNE1 – CUBN – SYNE2 – UBR4 – NEB –PKHD1 – OBSCN – DST
**KIRC**	TTN – PBRM1 – LRP2 – NEB – RNR2 – COL11A1 – HMCN1 – VHL – SYNE1 – SYNE2
**ACC**	NF1 – MUC16 – TTN – MUC19 – RYR1 – LRP1 – CSMD1 – ABCA13 – HMCN1 – LRP1B
**UCEC-UCS**	TTN – NEB – RYR2 – DMD – SYNE1 – MUC16 – CSMD3 – LRP1B – HMCN1 – DST
**CESC**	TTN – DMD – MUC16 – RYR2 – NEB – MUC19 – SNHG14 – CSMD3 – OBSCN – SYNE1
**OV**	TTN – MUC16 – SYNE1 – CSMD3 – MYHAS – LRP2 – UBR4 – RYR2 – PKHD1 – DST
**PRAD**	SYNE1 – TTN – LRP1B – SNHG14 – MUC16 – CSMD3 – HMCN1 – MYHAS – KMT2D – WASL
**SKCM**	TTN – MUC16 –SNHG14 – MYHAS – DNAH5 – MGAM – LRP1B – CSMD2 –DNAH9 – RYR1
**THCA**	TTN – SYNE1 – HMCN1 – MACF1 – KIAA1109 – TG –MUC16 –SNHG14 – UTP20 – MYHAS
**LUSC**	TTN – SNHG14 – CSMD3 – LRP1B –SYNE1 – MUC16 – RYR2 – USH2A – COL22A1 – SPTA1
**LUAD**	TTN – LRP1B – CSMD3 – SNHG14 – RYR2 – MUC16 – USH2A – SPTA1 – MYHAS – COL11A1

Table S11. Ten most frequently mutated genes in all cancer types analyzed

Ten most frequently mutated genes	#Mutation in all patients	Patients with the mutations (Out of 302)	Mean of mutations per patient (SD)	Patients with mutations and H-TMB level (Out of 43)	Mutations in H-TMB patients - Mean of mutations per high patient (SD)		Patients with mutations and L-TMB level (Out of 259)	Mutations in L-TMB patients - Mean of mutations per low patient (SD)	
**TTN**	208	90 (30%)	2,31 (5.6)	33 (77%)	145	4,39 (9)	57 (22%)	63	1,1 (0,3)
**DMD**	116	82 (27%)	1,41 (1.2)	29 (67%)	58	2 (1.8)	53 (20%)	58	1,09 (0.3)
**MUC16**	114	67 (22%)	1,70 (1.5)	27 (63%)	63	2,33 (2.1)	40 (15%)	51	1,27 (0.7)
**RYR2**	103	60 (20%)	1,72 (3)	26 (60%)	63	2,42 (4.5)	34 (13%)	40	1,18 (0.4)
**NEB**	101	62 (20%)	1,63 (1.8)	26 (60%)	60	2,31 (2.7)	36 (14%)	41	1,14 (0.4)
**MUC19**	98	60 (20%)	1,63 (1.8)	28 (65%)	60	2,14 (2.5)	32 (12%)	38	1,19 (0.5)
**SNHG14**	90	57 (19%)	1,58 (1.9)	20 (46%)	51	2,55 (3)	37 (14%)	39	1,05 (0.7)
**CSMD3**	88	66 (22%)	1,33 (0.9)	23 (53%)	42	1,83 (1.4)	43 (17%)	46	1,07 (0.3)
**OBSCN**	88	47 (15%)	1,87 (2)	21 (49%)	57	2,71 (2.7)	26 (10%)	31	1,19 (0.5)
**SYNE1**	88	51 (17%)	1,72 (1.9)	24 (56%)	59	2,46 (2.6)	27 (10%)	29	1,07 (0.3)

Table S12. Ten most frequently genes percentage, mean, standard deviation, fisher and welch test in Cervical squamous cell carcinoma and Endocervical adenocarcinoma with threshold 20 mut/Mb

Ten most frequently mutated genes	#mutation in all patients	Patients with the mutations (Out of 180)	Mean of mutations per patient (SD)	Patients with mutations and H-TMB level (Out of 20)	Mutations in high TMB patients - Mean of mutations per high patient (SD)		Patients with mutations and low TMB level (Out of 160)	Mutations in low TMB patients - Mean of mutations per low patient (SD)	
**TTN**	185	100 (55%)	1.85 (1.1)	19 (95%)	52	2.74 (1.4)	81 (51%)	133	1.64 (0.9)
**MUC19**	131	74 (41%)	1.77 (1)	17 (85%)	38	2.23 (1)	57 (36%)	93	1.63 (1)
**LRP1B**	128	79 (44%)	1.62 (1.1)	14 (70%)	32	2.28 (1.7)	65 (41%)	96	1.47 (0.9)
**CSMD3**	103	69 (38%)	1,49 (1)	12 (60%)	28	2,33 (1.9)	57 (36%)	75	1,31 (0.5)
**SNHG14**	102	64 (35%)	1,59 (1)	15 (75%)	35	2,33 (1.4)	49 (31%)	67	1,37 (0.7)
**SYNE1**	96	66 (37%)	1.45 (0.8)	15 (75%)	33	2.2 (1.1)	51 (32%)	63	1.23 (0.5)
**MUC16**	85	63 (35%)	1.35 (0.7)	13 (65%)	23	1.77 (1)	50 (31%)	62	1.24 (0.6)
**RYR2**	80	59 (33%)	1.35 (0.6)	15 (75%)	25	1.67 (0.9)	44 (27%)	55	1.25 (0.5)
**HMCN1**	79	62 (34%)	1,27 (0.6)	12 (60%)	18	1,5 (0.9)	50 (31%)	61	1,22 (0.5)
**COL11A1**	75	58 (32%)	1.29 (0.6)	9 (45%)	16	1.78 (0.8)	49 (31%)	59	1.20 (0.4)

Table S13. Ten most frequently genes percentage, mean, standard deviation, fisher and welch test in Esophageal carcinoma with threshold 20 mut/Mb

Ten most frequently mutated genes	#mutation in all patients	Patients with the mutations	Mean of mutations per patient	Patients with mutations and high TMB level (Out of 38)	Number of mutations in high TMB patients - Mean of mutations per high patient (SD)		Patients with mutations and low TMB level (Out of 181)	Number of mutations in low TMB patients - Mean of mutations per low patient (SD)	
**TTN**	273	111 (51%)	2.46 (1.9)	35 (92%)	132	3.77 (2.3)	76 (42%)	141	1.85 (1.3)
**MUC16**	166	96 (44%)	1.73 (1)	27 (71%)	59	2.18 (1.2)	69 (38%)	107	1.55 (0.8)
**SYNE1**	95	64 (29%)	1.48 (1)	22 (58%)	41	1.86 (1.3)	42 (23%)	54	1.28 (0.7)
**CSMD3**	84	64 (29%)	1,31 (0.6)	18 (47%)	26	1,44 (0.6)	46 (25%)	58	1,26 (0.6)
**MYHAS**	82	64 (29%)	1,28 (0.6)	23 (60%)	38	1,65 (0.9)	41 (23%)	44	1,07 (0.3)
**LRP2**	79	63 (29%)	1,25 (0.5)	18 (47%)	27	1,5 (0.7)	45 (25%)	52	1,15 (0.4)
**UBR4**	79	56 (25%)	1,41 (0.9)	20 (53%)	31	1,55 (1.2)	36 (20%)	48	1,33 (0.6)
**RYR2**	76	63 (29%)	1.21 (0.5)	18 (47%)	21	1.67 (0.4)	45 (25%)	55	1.22 (0.5)
**PKHD1**	74	61 (28%)	1,21 (0.5)	23 (60%)	31	1,35 (0.6)	38 (21%)	43	1,13 (0.3)
**DST**	73	54 (25%)	1.35 (0.6)	15 (39%)	22	1.47 (0.8)	39 (21%)	51	1.31 (0.6)

Table S14. Ten most frequently genes percentage, mean, standard deviation, fisher and welch test in Ovarian serous cystadenocarcinoma in threshold 20 mut/Mb

Ten most frequently mutated genes	#mutation in all patients	Patients with the mutations (Out of 540)	Mean of mutations per patient (SD)	Patients with mutations and H-TMB level (Out of 227)	Number of mutations in high TMB patients - Mean of mutations per high patient (SD)		Patients with mutations and low TMB level (Out of 313)	Number of mutations in ow TMB patients - Mean of mutations per low patient (SD)	
**TTN**	2745	265 (49%)	10,36 (17.2)	197 (87%)	2667	13,54 (18.9)	68 (22%)	78	1,15 (0.4)
**NEB**	1503	217 (40%)	6,93 (9.5)	170 (75%)	1445	8,5 (10.2)	47 (15%)	58	1,23 (0.5)
**RYR2**	1383	233 (43%)	5,93 (8.6)	175 (77%)	1315	7,51 (9.4)	58 (18%)	68	1,17 (0.4)
**DMD**	1374	216 (40%)	6,36 (9)	169 (74%)	1321	7,82 (9.6)	47 (15%)	53	1,13 (0.3)
**SYNE1**	1333	216 (40%)	6,17 (9)	173 (76%)	1286	7,43 (9.8)	43 (14%)	47	1,09 (0.3)
**MUC16**	1177	193 (36%)	6,1 (8)	153 (67%)	1126	7,36 (8.6)	40 (13%)	51	1,27 (0.7)
**CSMD3**	1141	215 (40%)	5,31 (7.7)	165 (73%)	1084	6,57 (8.4)	50 (16%)	57	1,14 (0.5)
**LRP1B**	1088	189 (35%)	5,76 (7.7)	150 (66%)	1045	6,97 (8.2)	39 (12%)	43	1,1 (0.3)
**HMCN1**	1080	193 (53%)	5,59 (7.2)	160 (70%)	1042	6,51 (7.7)	33 (10%)	38	1,15 (0.4)
**DST**	1056	181 (33%)	5,83 (8.1)	153 (67%)	1024	6,69 (8.6)	28 (9%)	32	1,14 (0.4)

Table S15. Ten most frequently genes percentage, mean, standard deviation, fisher and welch test in Uterine Corpus Endometrial Carcinoma and Uterine Carcinosarcoma with threshold 20 mut/Mb

Ten most frequently mutated genes	#mutation in all patients	Patients with the mutations (Out of 415)	Mean of mutations per patient) (SD)	Patients with mutations and H-TMB level (Out of 84)	Number of mutations in high TMB patients - Mean of mutations per high patient (SD)		Patients with mutations and low TMB level (Out of 331)	Number of mutations in ow TMB patients - Mean of mutations per low patient (SD)	
**TTN**	387	176 (42%)	2,2 (2.2)	71 (84%)	216	3,04 (3.1)	105 (32%)	171	1,63 (1.1)
**SYNE1**	308	141 (34%)	2,18 (1.8)	65 (77%)	199	3,06 (2.3)	76 (23%)	109	1,43 (0.8)
**LRP1B**	266	154 (37%)	1,73 (1.4)	58 (69%)	124	2,14 (2.1)	96 (29%)	142	1,47 (0.7)
**SNHG14**	260	139 (33%)	1,87 (1.3)	63 (75%)	158	2,51 (1.5)	76 (23%)	102	1,34 (0.6)
**CSMD3**	248	136 (33%)	1,82 (1.7)	58 (69%)	150	2,59 (2.3)	78 (23%)	98	1,26 (0.6)
**MYHAS**	189	99 (24%)	1,91 (1.7)	57 (68%)	141	2,47 (2)	42 (13%)	48	1,14 (0.3)
**HMCN1**	186	117 (28%)	1,59 (1.5)	54 (64%)	114	2,11 (2.1)	63 (19%)	72	1,14 (0.4)
**MUC16**	167	144 (27%)	1,46 (1.1)	48 (57%)	87	1,81 (1.5)	66 (20%)	80	1,21 (0.5)
**DNAH5**	165	97 (23%)	1,7 (1.4)	52 (62%)	112	2,15 (1.8)	45 (13%)	53	1,18 (0.4)
**SPTA1**	158	110 (26%)	1,44 (1)	43 (51%)	68	1,58 (1.3)	67 (20%)	90	1,34 (0.7)

Table S16. Ten most frequently genes percentage, mean, standard deviation, fisher and welch test in Stomach adenocarcinoma with threshold 20 mut/Mb

Ten most frequently mutated genes	#mutation in all patients	Patients with the mutations (Out of 398)	Mean of mutations per patient (SD)	Patients with mutations and high TMB level (Out of 12)	Number of mutations in high TMB patients - Mean of mutations per high patient (SD)		Patients with mutations and low TMB level (Out of 386)	Number of mutations in low TMB patients - Mean of mutations per low patient (SD)	
**ALB**	187	133 (33%)	1.41 (0.8)	5 (42%)	12	2.4 (1.1)	128 (33%)	175	1.37 (0.8)
**TTN**	150	109 (27%)	1.38 (0.7)	9 (75%)	18	2 (1.6)	100 (26%)	132	1.32 (0.6)
**CSMD3**	121	86 (22%)	1.41 (0.8)	7 (58%)	18	2.57 (1.9)	79 (20%)	103	1.3 (0.6)
**RYR2**	117	90 (23%)	1.3 (0.7)	8 (67%)	20	2.5 (1.2)	82 (21%)	97	1.18 (0.4)
**MUC19**	112	87 (22%)	1.28 (0.7)	9 (75%)	19	2.11 (1.6)	78 (20%)	93	1.19 (0.4)
**OBSCN**	187	133 (33%)	1.4 (0.8)	5 (42%)	12	2.4 (1.1)	128 (33%)	175	1.4 (0.8)
**HMCN1**	101	70 (17%)	1.44 (1)	7 (58%)	23	3.28 (2.3)	63 (16%)	78	1.24 (0.5)
**COL11A1**	96	76 (19%)	1.26 (0.7)	7 (58%)	17	2.43 (1.6)	69 (18%)	79	1.14 (0.4)
**MUC16**	96	79 (20%)	1.21 (0.5)	8 (67%)	14	1.75 (0.9)	71 (18%)	82	1.15 (0.4)
**LRP1B**	95	76 (19%)	1.25 (0.7)	7 (58%)	15	2.14 (1.8)	69 (18%)	80	1.16 (0.4)

Table S17. Ten most frequently genes percentage, mean, standard deviation, fisher and welch test in Liver hepatocellular carcinoma with threshold 20 mut/Mb

Ten most frequently mutated genes	#mutation in all patients	Patients with the mutations (Out of 496)	Mean of mutations per patient (SD)	Patients with mutations and high TMB level (Out of 2)	Number of mutations in high TMB patients - Mean of mutations per high patient (SD)		Patients with mutations and low TMB level (Out of 494)	Number of mutations in low TMB patients - Mean of mutations per low patient (SD)	
**SYNE1**	55	38 (8%)	1.45 (2.6)	1 (50%)	17	17 (NA)	37 (7%)	38	1.03 (0.2)
**TTN**	54	33 (7%)	1.64 (3.1)	2 (100%)	21	10.5 (12)	31 (6%)	33	1.06 (0.2)
**LRP1B**	41	31 (6%)	1.32 (1.4)	2 (100%)	10	5 (5.6)	29 (6%)	31	1.07 (0.3)
**SNHG14**	37	25 (5%)	1.48 (2.2)	2 (100%)	13	6.5 (7.8)	23 (5%)	24	1.04 (0.2)
**MUC16**	35	22 (4%)	1.59 (1.9)	2 (100%)	11	5.5 (6.4)	20 (4%)	24	1.2 (0.4)
**CSMD3**	32	30 (6%)	1.07 (0.4)	1 (50%)	3	3 (NA)	29 (6%)	29	1 (0)
**HMCN1**	30	27 (5%)	1.11 (0.4)	2 (100%)	4	2 (1.4)	25 (5%)	26	1.04 (0.2)
**MYHAS**	30	21 (4%)	1.43 (1.1)	2 (100%)	7	3.5 (3.5)	19 (4%)	23	1.21 (0.4)
**KMT2D**	27	22 (4%)	1.23 (0.7)	1 (50%)	4	4 (NA)	21 (4%)	23	1.09 (0.3)
**WASL**	26	25 (5%)	1.04 (0.2)	0 (0%)	0	0 (0)	25 (5%)	26	1.04 (0.2)

Table S18. Ten most frequently genes percentage, mean, standard deviation, fisher and welch test in Prostate adenocarcinoma with threshold 20 mut/Mb

Ten most frequently mutated genes	#mutation in all patients	Patients with the mutations (Out of 408)	Mean of mutations per patient (SD)	Patients with mutations and high TMB level (Out of 46)	Number of mutations in high TMB patients - Mean of mutations per high patient (SD)		Patients with mutations and low TMB level (Out of 362)	Number of mutations in low TMB patients - Mean of mutations per low patient (SD)	
**TTN**	212	130 (32%)	1,63 (1.4)	32 (69%)	80	2,5 (2.5)	98 (27%)	132	1,35 (0,41)
**SNHG14**	127	98 (24%)	1,29 (0.7)	28 (61%)	48	1,71 (1.2)	70 (19%)	79	1,13 (0,11)
**SYNE1**	126	82 (20%)	1,54 (2)	24 (52%)	55	2,29 (3.5)	58 (16%)	71	1,22 (0,25)
**CSMD3**	102	73 (18%)	1,4 (1.5)	17 (37%)	41	2,41 (3)	56 (15%)	61	1,09 (0,08)
**HMCN1**	101	81 (20%)	1,25 (0.6)	20 (43%)	30	1,5 (0.9)	61 (17%)	71	1,16 (0,21)
**MYHAS**	97	71 (17%)	1,37 (1.1)	14 (30%)	29	2,07 (2.2)	57 (16%)	68	0,37 (1,19)
**MUC16**	96	72 (18%)	1,33 (0.8)	18 (39%)	35	1,94 (1.2)	54 (15%)	61	1,13 (0,15)
**RYR2**	95	75 (37%)	1,27 (0.8)	23 (100%)	34	1,48 (1.3)	52 (29%)	61	1,17 (0,18)
**LRP1B**	90	68 (17%)	1,32 (1)	17 (37%)	31	1,82 (1.9)	51 (14%)	59	1,16 (0,13)
**SYNE2**	89	65 (16%)	1,37 (0.7)	23 (50%)	40	1,74 (0.8)	42 (12%)	49	0,29 (1,67)

Table S19. Ten most frequently genes percentage, mean, standard deviation, fisher and welch test in Bladder Urothelial Carcinoma with threshold 20 mut/Mb

Ten most frequently mutated genes	#mutation in all patients	Patients with the mutations (Out of 176)	Mean of mutations per patient (SD)	Patients with mutations and high TMB level (Out of 4)	Number of mutations in high TMB patients - Mean of mutations per high patient (SD)		Patients with mutations and low TMB level (Out of 172)	Number of mutations in low TMB patients - Mean of mutations per low patient (SD)	
**TTN**	91	22 (12%)	4,14 (11.7)	4 (100%)	70	17,5 (25.8)	18 (10%)	21	1,17 (0.4)
**SYNE1**	48	15 (8%)	3,2 (7.7)	4 (100%)	37	9,25 (14.6)	11 (6%)	11	1 (0)
**MUC16**	45	15 (8%)	3 (7.5)	2 (50%)	32	16 (19.8)	13 (7%)	13	1 (0)
**HMCN1**	37	11 (6%)	3,36 (7.2)	3 (75%)	28	9,33 (13.6)	8 (5%)	9	1,12 (0.3)
**LRP1B**	37	18 (10%)	2,05 (3.8)	4 (100%)	22	5,5 (7.7)	14 (8%)	15	1,07 (0.03)
**SNHG14**	36	15 (8%)	2,4 (4.9)	4 (100%)	24	6 (9.3)	11 (6%)	12	1,1 (0.3)
**MACF1**	35	9 (5%)	3,89 (6.1)	4 (100%)	30	7,5 (8.3)	5 (3%)	5	1 (0)
**RYR3**	35	14 (8%)	2,5 (4)	3 (75%)	23	7,67 (7.2)	11 (6%)	12	1,09 (0.3)
**USH2A**	34	13 (7%)	2,61 (5)	4 (100%)	24	6 (8.7)	9 (5%)	10	1,11 (0.33)
**NEB**	33	11 (6%)	3 (6.3)	4 (100%)	26	6,5 (10.3)	7 (4%)	7	1 (0)

Table S20. Ten most frequently genes percentage, mean, standard deviation, fisher and welch test in Pancreatic adenocarcinoma with threshold 20 mut/Mb

Ten most frequently mutated genes	#mutation in all patients	Patients with the mutations (Out of 494)	Mean of mutations per patient (SD)	Patients with mutations and high TMB level (Out of 19)	Number of mutations in high TMB patients - Mean of mutations per high patient (SD)		Patients with mutations and low TMB level (Out of 475)	Number of mutations in low TMB patients - Mean of mutations per low patient (SD)	
**TTN**	1055	341 (69%)	3.09 (2.3)	19 (100%)	135	7.1 (3.2)	322 (68%)	920	2.86 (2)
**SNHG14**	585	296 (60%)	1.98 (1.5)	15 (79%)	63	4.2 (3.4)	281 (59%)	522	1.86 (1.2)
**CSMD3**	473	259 (52%)	1.82 (1.3)	16 (84%)	59	3.69 (3.3)	243 (51%)	414	1.7 (1)
**LRP1B**	468	254 (51%)	1.84 (1.3)	17 (89%)	59	3.47 (2.7)	237 (50%)	409	1.72 (1)
**SYNE1**	410	225 (45%)	1.82 (1.1)	18 (95%)	53	2.94 (1.7)	207 (43%)	357	1.72 (1)
**MUC16**	384	209 (42%)	1.84 (1.2)	17 (89%)	54	3.18 (2.3)	192 (40%)	330	1.72 (1)
**RYR2**	367	224 (45%)	1.64 (1)	15 (79%)	44	2.93 (1.6)	209 (44%)	323	1.54 (0.9)
**USH2A**	327	214 (43%)	1.53 (0.8)	16 (84%)	29	1.81 (0.8)	198 (42%)	298	1.5 (0.8)
**COL22A1**	285	189 (38%)	1.51 (1)	11 (58%)	33	3 (1.6)	178 (37%)	252	1.41 (0.8)
**SPTA1**	282	191 (39%)	1.48 (0.8)	13 (68%)	25	1.92 (1)	178 (37%)	257	1.44 (0.7)

Table S21. Ten most frequently genes percentage, mean, standard deviation, fisher and welch test in Lung Squamous Cell Cancer with threshold 20 mut/Mb

Ten most frequently mutated genes	#mutation in all patients	Number and percentage of patients with the mutations (Out of 512)	Mean of mutations per patient (SD)	Number and percentage of patients with mutations and high TMB level (Out of 29)	Number of mutations in high TMB patients - Mean of mutations per high patient (SD)		Number and percentage of patients with mutations and low TMB level (Out of 483)	Number of mutations in low TMB patients - Mean of mutations per low patient (SD)	
**TTN**	531	199 (39%)	2.67 (2.7)	29 (100%)	190	6.55 (4.7)	170 (35%)	341	2 (1.3)
**LRP1B**	481	231 (45%)	2.08 (1.7)	27 (93%)	110	4.07 (3)	204 (42%)	371	1.82 (1.3)
**CSMD3**	455	225 (44%)	2.02 (1.6)	27 (93%)	112	4.15 (2.7)	198 (41%)	343	1.73 (1)
**SNHG14**	441	224 (44%)	1.97 (1.4)	25 (86%)	83	3.32 (2.2)	199 (41%	358	1.8 (1.2)
**RYR2**	439	209 (41%)	2.1 (1.6)	29 (100%)	114	3.93 (3)	180 (37%)	325	1.8 (1)
**MUC16**	365	171 (33%)	2.13 (1.4)	27 (93%)	105	3.89 (2)	144 (30%)	260	1.8 (1)
**USH2A**	342	179 (35%)	1.91 (1.4)	25 (86%)	98	3.92 (2.4)	154 (32%)	244	1.58 (0.9)
**SPTA1**	305	174 (34%)	1.75 (1.1)	26 (90%)	75	2.88 (1.6)	148 (31%)	230	1.55 (0.8)
**MYHAS**	248	167 (33%)	1.48 (0.9)	19 (65%)	33	1.74 (0.9)	148 (31%)	215	1.45 (0.9)
**COL11A1**	229	144 (28%)	1.59 (0.9)	25 (86%)	62	2.48 (1.3)	119 (25%)	167	1.4 (0.6)

Table S22. Ten most frequently genes percentage, mean, standard deviation, fisher and welch test in Lung Adenocarcinoma with threshold 20 mut/Mb

Ten most frequently mutated genes	#mutation in all patients	Number and percentage of patients with the mutations (Out of 494)	Mean of mutations per patient (SD)	Number and percentage of patients with mutations and high TMB level (Out of 13)	Number of mutations in high TMB patients - Mean of mutations per high patient (SD)		Number and percentage of patients with mutations and low TMB level (Out of 481)	Number of mutations in L-TMB patients - Mean of mutations per low patient (SD)	
**TTN**	105	33 (7%)	3.18 (7.3)	11 (85%)	77	7 (12)	22 (4%)	28	1.27 (0.5)
**SYNE1**	57	27 (5%)	2.11 (2.7)	8 (61%)	34	4.25 (4.4)	19 (4%)	23	1.21 (0.4)
**HMCN1**	56	23 (5%)	2.43 (3.7)	9 (69%)	37	4.11 (5.5)	14 (3%)	19	1.36 (0.8)
**MACF1**	47	22 (4%)	2.14 (3.2)	7 (54%)	28	4 (5.4)	15 (3%)	19	1.27 (0.6)
**KIAA1109**	45	19 (4%)	2.37 (2.1)	11 (85%)	36	3.27 (2.4)	8 (2%)	9	1.12 (0.3)
**TG**	45	39 (8%)	1.15 (0.5)	4 (31%)	6	1.5 (1)	35 (7%)	39	1.11 (0.4)
**MUC16**	44	17 (3%)	2.59 (4.1)	10 (77%)	37	3.7 (5.1)	7 (1%)	7	1 (0)
**SNHG14**	40	25 (5%)	1.6 (1.5)	7 (54%)	19	2.71 (2.5)	18 (4%)	21	1.67 (0.5)
**UTP20**	38	16 (3%)	2.37 (2.6)	10 (77%)	30	3 (3.2)	6 (1%)	8	1.33 (0.5)
**MYHAS**	36	17 (3%)	2.12 (2.5)	11 (85%)	30	2.72 (3)	6 (1%)	6	1 (0)

Table S23. Ten most frequently genes percentage, mean, standard deviation, fisher and welch test in Thyroid carcinoma with threshold 20 mut/Mb

Ten most frequently mutated genes	#mutation in all patients	Patients with the mutations (Out of 227)	Mean of mutations per patient (SD)	Patients with mutations and high TMB level (Out of 3)	Number of mutations in high TMB patients - Mean of mutations per high patient (SD)		Patients with mutations and low TMB level (Out of 224)	Number of mutations in low TMB patients - Mean of mutations per low patient (SD)	
**MUC16**	21	17 (7%)	1.23 (0.6)	3 (100%)	6	2 (1)	14 (6%)	15	1.07 (0.3)
**TTN**	21	17 (7%)	1.23 (0.7)	2 (67%)	6	3 (1.4)	15 (7%)	15	1 (0)
**MUC19**	16	12 (5%)	1.33 (0.6)	3 (100%)	4	1.33 (0.6)	9 (4%)	12	1.33 (0.7)
**RYR1**	16	10 (4%)	1.6 (0.7)	3 (100%)	7	2.33 (0.6)	7 (3%)	9	1.28 (0.5)
**LRP1**	15	9 (4%)	1.7 (1)	1 (33%)	4	4 (NA)	8 (3%)	11	1.4 (0.5)
**CSMD1**	14	11 (5%)	1.27 (0.6)	2 (67%)	4	2 (1.4)	9 (4%)	10	1.11 (0.3)
**ABCA13**	13	12 (5%)	1.08 (0.3)	2 (67%)	3	1.5 (0.7)	10 (4%)	10	1 (0)
**HMCN1**	13	12 (5%)	1.08 (0.3)	2 (67%)	2	1 (0)	10 (4%)	11	1.1 (0.3)
**LRP1B**	13	10 (4%)	1.3 (0.7)	2 (67%)	4	2 (1.4)	8 (3%)	9	1.12 (0.3)

Table S24. Ten most frequently genes percentage, mean, standard deviation, fisher and welch test in Adrenocortical carcinoma with threshold 20 mut/Mb

Ten most frequently mutated genes	#mutations in all patients	Patients with the mutations (Out of 338)	Mean of mutations per patient (SD)	Patients with mutations and high TMB level (Out of 3)	Mutations in high TMB patients - Mean of mutations per high patient (SD)		Patients with mutations and low TMB level (Out of 335)	Number of mutations in low TMB patients - Mean of mutations per low patient (SD)	
**TTN**	74	48 (14%)	1.54 (2.3)	3 (100%)	20	6.67 (9)	45 (12%)	54	1.2 (0.6)
**PBRM1**	64	61 (18%)	1.05 (0.2)	1 (33%)	2	2 NA	60 (18%)	62	1.03 (0.2)
**LRP2**	50	43 (13%)	1.16 (0.5)	2 (67%)	4	2 (1.4)	41 (12%)	46	1.12 (0.4)
**NEB**	39	27 (8%)	1.44 (1.9)	3 (100%)	14	4.67 (5.5)	24 (7%)	25	1.04 (0.2)
**RNR2**	38	37 (11%)	1.03 (0.2)	0 (0%)	0	0 (0)	37 (11%)	38	1.03 (0.2)
**COL11A1**	37	30 (9%)	1.23 (0.9)	1 (33%)	6	6 (NA)	29 (9%)	31	1.07 (0.3)
**HMCN1**	37	26 (8%)	1.42 (1.2)	2 (67%)	8	4 (4.2)	24 (7%)	29	1.21 (0.5)
**VHL**	37	37 (11%)	1 (0)	0 (0%)	0	0 (0)	37 (11%)	37	1 (0)
**SYNE1**	36	30 (9%)	1.2 (0.8)	1 (33%)	5	5 (NA)	29 (9%)	31	1.07 (0.3)
**SYNE2**	33	28 (8%)	1.18 (0.6)	2 (67%)	5	2.5 (2.1)	26 (8%)	28	1.08 (0.3)

Table S25. Ten most frequently genes percentage, mean, standard deviation, fisher and welch test in Kidney renal clear cell carcinoma with threshold 20 mut/Mb

Ten most frequently mutated genes	#mutation in all patients	Patients with the mutations (Out of 287)	Mean of mutations per patient (SD)	Patients with mutations and high TMB level (Out of 4)	Number of mutations in high TMB patients - Mean of mutations per high patient (SD)		Patients with mutations and low TMB level (Out of 283)	Number of mutations in low TMB patients - Mean of mutations per low patient (SD)	
**TTN**	87	66 (23%)	1.32 (0.7)	4 (100%)	10	2.5 (1.3)	62 (22%)	77	1.24 (0.6)
**LRP2**	62	51 (18%)	1.21 (0.5)	3 (70%)	5	1.67 (1.1)	48 (17%)	57	1.19 (0.4)
**SYNE1**	48	36 (12%)	1.33 (0.5)	4 (100%)	5	1.25 (0.5)	32 (11%)	43	1.34 (0.5)
**CUBN**	44	37 (13%)	1.19 (0.4)	1 (25%)	2	2 NA	36 (13%)	42	1.17 (0.4)
**SYNE2**	44	39 (13%)	1.13 (0.5)	3 (75%)	6	2 (1.7)	36 (13%)	38	1.05 (0.2)
**UBR4**	41	35 (12%)	1.17 (0.4)	2 (50%)	3	1.5 (0.70)	33 (12%)	38	1.15 (0.4)
**NEB**	36	31 (11%)	1.16 (0.4)	2 (50%)	2	1 (0)	29 (10%)	34	1.17 (0.4)
**PKHD1**	36	33 (11%)	1.09 (0.3)	2 (50%)	2	1 (0)	31 (11%)	34	1.1 (0.3)
**OBSCN**	35	33 (11%)	1.06 (0.2)	3 (75%)	5	1.67 (0.6)	30 (11%)	30	1 (0)
**DST**	32	29 (10%)	1.1 (0.3)	1 (25%)	2	2 (NA)	28 (10%)	30	1.07 (0.3)

Table S26. Ten most frequently genes percentage, mean, standard deviation, fisher and welch test in Kidney renal papillary cell carcinoma with threshold 20 mut/Mb

Ten most frequently mutated genes	#mutation in all patients	Patients with the mutations (Out of 465)	Mean of mutations per patient (SD)	Patients with mutations and high TMB level (Out of 256)	Mutations in high TMB patients - Mean of mutations per high patient (SD)		Number and percentage of patients with mutations and low TMB level (Out of 209)	Mutations in low TMB patients - Mean of mutations per low patient (SD)	
**TTN**	1877	350 (75%)	5,36 (7.2)	239 (93%)	1685	7,05 (8.2)	111 (53%)	192	1,73 (1)
**MUC16**	1058	286 (62%)	3,7 (4.8)	210 (82%)	951	4,53 (5.3)	76 (36%)	107	1,41 (0.7)
**SNHG14**	856	286 (61%)	2,99 (3.4)	208 (81%)	737	3,54 (3.8)	78 (37%)	119	1,52 (0.8)
**MYHAS**	793	266 (57%)	2,98 (3.4)	208 (81%)	713	3,43 (3.7)	58 (28%)	80	1,38 (0.6)
**DNAH5**	773	269 (58%)	2,87 (3.2)	203 (79%)	686	3,38 (3.5)	66 (31%)	87	1,32 (0.6)
**MGAM**	745	280 (60%)	2,66 (3.1)	203 (79%)	637	3,14 (3.5)	77 (37%)	108	1,4 (0.7)
**LRP1B**	600	234 (50%)	2,56 (3.2)	181 (71%)	535	2,95 (3.5)	53 (25%)	65	1,23 (0.6)
**CSMD2**	546	236 (51%)	2,31 (2.5)	184 (72%)	482	2,62 (2.7)	52 (25%)	64	1,23 (0.6)
**DNAH9**	449	206 (44%)	2,18 (2.7)	163 (64%)	392	2,4 (3)	43 (20%)	57	1,32 (0.7)
**RYR1**	448	213 (46%)	2,1 (2.4)	160 (62%)	385	2,41 (2.7)	53 (25%)	63	1,19 (0.4)

Table S27. Ten most frequently genes percentage, mean, standard deviation, fisher and welch test in Skin Cutaneous Melanoma with threshold 20 mut/Mb
